# Supplementary material for: Reprogramming insulin receptor activation with a de novo agonist to overcome severe insulin resistance
Source: bioRxiv. 2026 May 7:2026.05.04.722722. Preprint. [Version 1] doi: 10.64898/2026.05.04.722722 (PMC13174351; doi:10.64898/2026.05.04.722722)
Supplement: 1 [file NIHPP2026.05.04.722722V1-supplement-1.pdf]

bioRxiv preprint doi: https://doi.org/10.64898/2026.05.04.722722; this version posted May 7, 2026. The copyright holder for this preprint (which was not certified by peer review) is the author/funder, who has granted bioRxiv a license to display the preprint in perpetuity. It is made available under aCC-BY 4.0 International license.

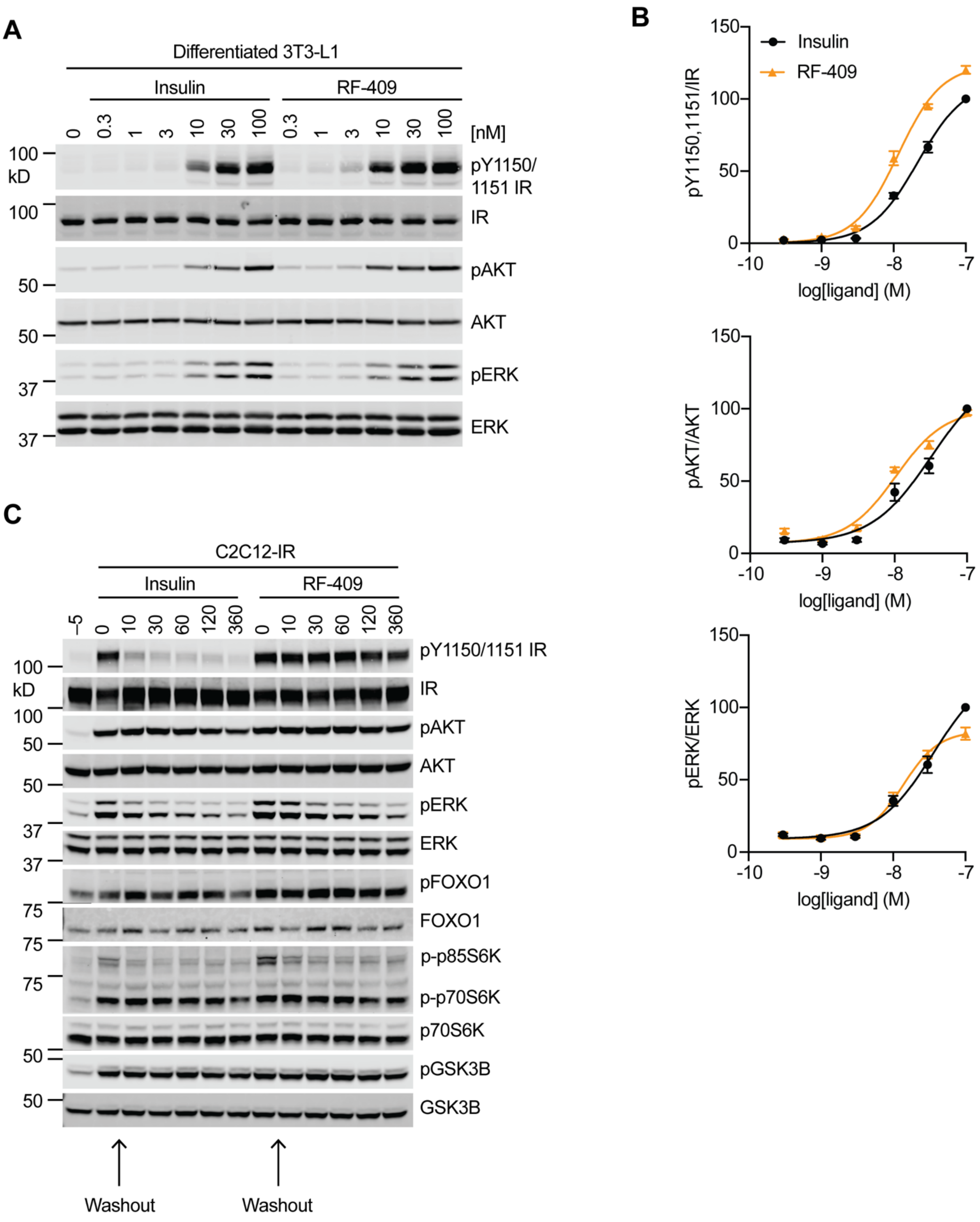

Fig. S1. RF-409 potently activates and prolongs IR signaling.

(A) Representative immunoblot of differentiated 3T3-L1 adipocytes fasted for 12 h and treated with the indicated concentrations of insulin or RF-409 for 10 min.

bioRxiv preprint doi: https://doi.org/10.64898/2026.05.04.722722; this version posted May 7, 2026. The copyright holder for this preprint (which was not certified by peer review) is the author/funder, who has granted bioRxiv a license to display the preprint in perpetuity. It is made available under aCC-BY 4.0 International license.

(B) Quantification of immunoblot data shown in (A), fit by nonlinear regression. Phosphorylation levels were normalized to total protein and expressed relative to the response to 100 nM insulin. Data are presented as mean ± SEM; $n = 3$ independent experiments.

(C) Representative immunoblot of C2C12-IR cells fasted for 4 h and treated with insulin or RF-409 (10 nM) for 5 min, followed by ligand washout and incubation for the indicated time points.

bioRxiv preprint doi: https://doi.org/10.64898/2026.05.04.722722; this version posted May 7, 2026. The copyright holder for this preprint (which was not certified by peer review) is the author/funder, who has granted bioRxiv a license to display the preprint in perpetuity. It is made available under aCC-BY 4.0 International license.

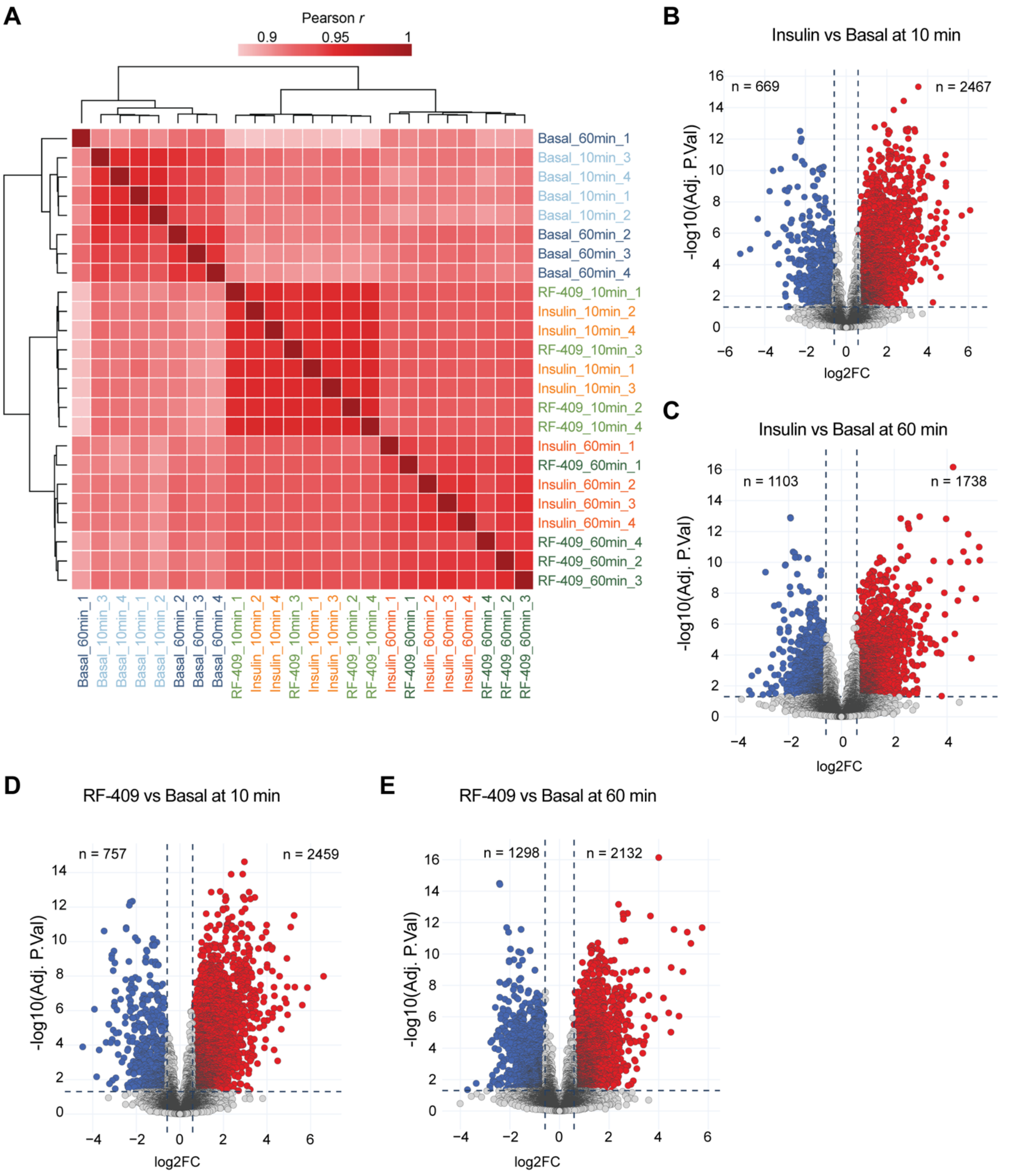

1443

1444 **Fig. S2. Phosphoproteomic signatures of insulin and RF-409.**

1445 **(A)** Hierarchically clustered heatmap of phosphoproteomic profiles across basal, insulin-, and RF-409–
1446 treated conditions at 10 and 60 min.

1447 **(B–E)** Volcano plots showing phosphosites significantly regulated after 10 or 60 min of insulin or RF-409
1448 treatment relative to basal conditions.

bioRxiv preprint doi: https://doi.org/10.64898/2026.05.04.722722; this version posted May 7, 2026. The copyright holder for this preprint (which was not certified by peer review) is the author/funder, who has granted bioRxiv a license to display the preprint in perpetuity. It is made available under aCC-BY 4.0 International license.

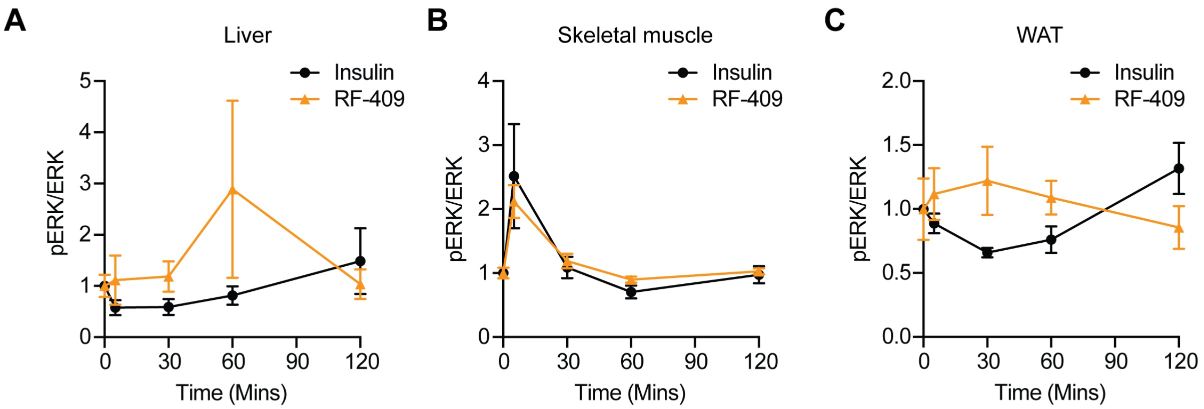

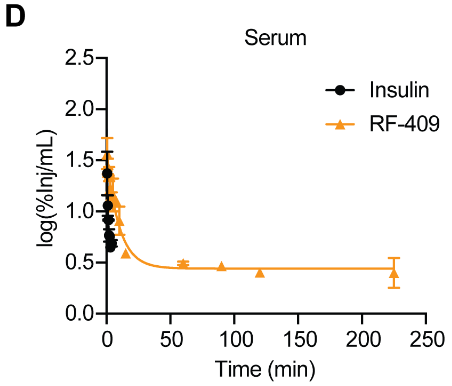

Ligand	Regression type	Slope (log(%Inj/ml)/min)	Half-life (min)
Insulin	Full (0.5–3 min)	–0.30493	0.99
	Terminal (1.5–3 min)	–0.16365	1.84
RF-409	Full (0.5–225 min)	–0.00518	58.1
	Terminal (≥ 60 min)	–0.0005415	555.9 (~9.3 h)

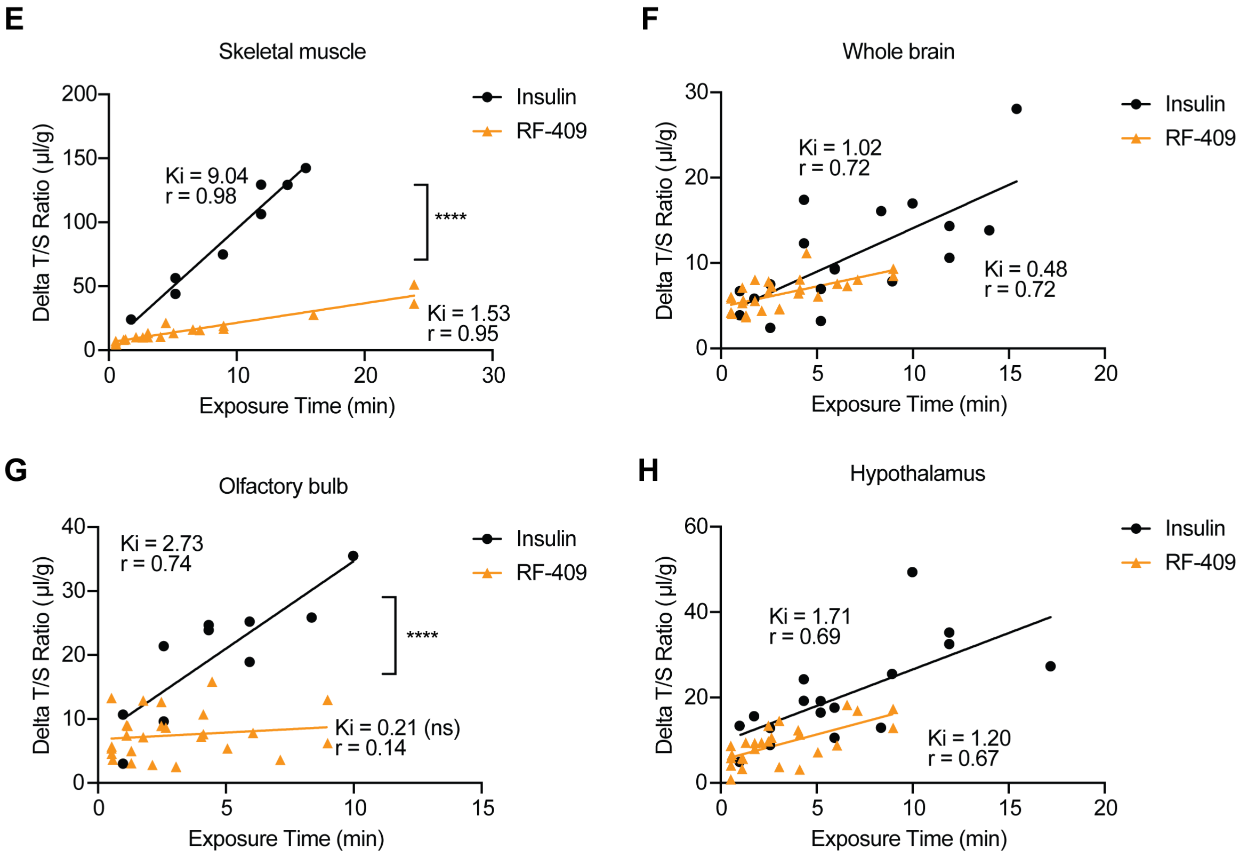

Fig. S3. RF-409 exhibits extended stability in vivo and limited tissue penetration.

bioRxiv preprint doi: https://doi.org/10.64898/2026.05.04.722722; this version posted May 7, 2026. The copyright holder for this preprint (which was not certified by peer review) is the author/funder, who has granted bioRxiv a license to display the preprint in perpetuity. It is made available under aCC-BY 4.0 International license.

(A–C) Pharmacodynamic analysis of ERK phosphorylation (pERK) in liver (A), skeletal muscle (B), and epididymal white adipose tissue (eWAT) (C) following administration of insulin or RF-409, as described in Fig. 3A.

(D) Extended pharmacokinetic analysis of insulin and RF-409 in serum, with summary parameters.

(E–H) Tissue/serum ratios of insulin and RF-409 in skeletal muscle (E), whole brain (F), olfactory bulb (G), and hypothalamus (H) are plotted against exposure time. The slopes of the lines for each linear regression represent the unidirectional influx rate (Ki) in units of µl/g-min while the correlation coefficient is represented by ‘r’.

bioRxiv preprint doi: https://doi.org/10.64898/2026.05.04.722722; this version posted May 7, 2026. The copyright holder for this preprint (which was not certified by peer review) is the author/funder, who has granted bioRxiv a license to display the preprint in perpetuity. It is made available under aCC-BY 4.0 International license.

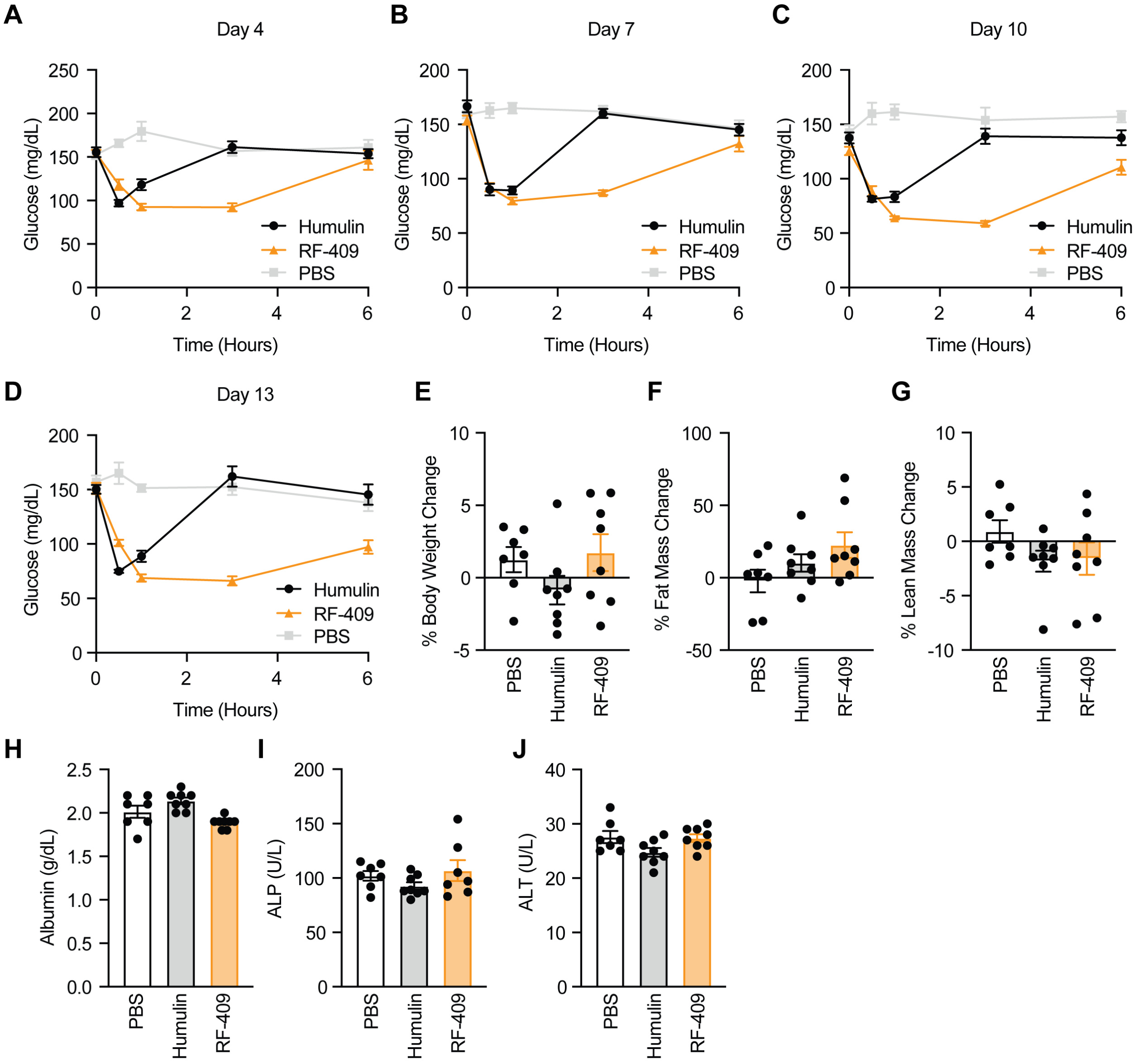

Fig. S4. RF-409 produces sustained glucose-lowering effects in vivo.

(A–D) Ad libitum tolerance tests performed on treatment days 4 (A), 7 (B), 10 (C), and 13 (D) in mice treated with insulin or RF-409, as described in Fig. 3G.

(E) Percent change in body weight over the treatment period.

(F) Percent change in fat mass.

(G) Percent change in lean mass.

(H) Plasma albumin levels.

(I) Plasma alkaline phosphatase (ALP) levels.

(J) Plasma alanine aminotransferase (ALT) levels.

Data are presented as mean ± SEM; *n* = 8 (RF-409 and Humulin) and n = 7 (PBS).

bioRxiv preprint doi: https://doi.org/10.64898/2026.05.04.722722; this version posted May 7, 2026. The copyright holder for this preprint (which was not certified by peer review) is the author/funder, who has granted bioRxiv a license to display the preprint in perpetuity. It is made available under aCC-BY 4.0 International license.

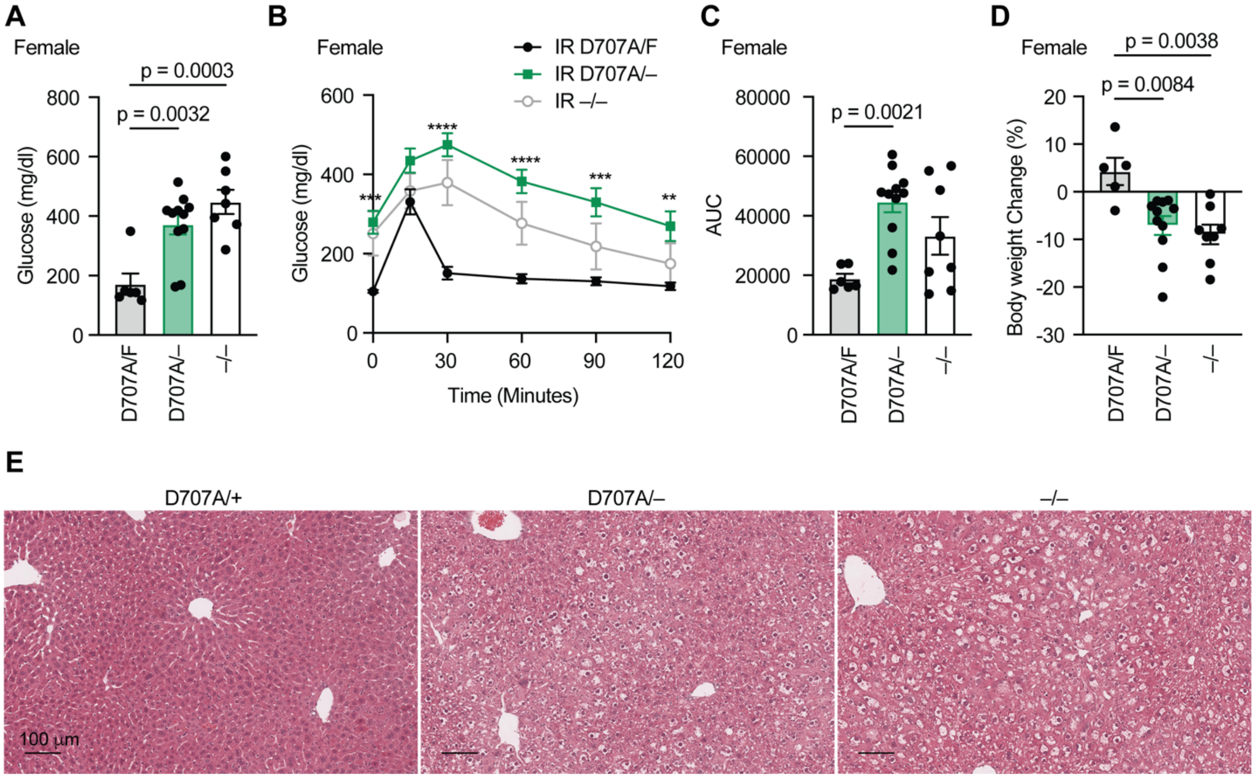

Fig. S5. Female and male IR D707A mice exhibit severe insulin resistance.

(A) Ad libitum blood glucose levels. D707A/F, n = 6; D707A/–, n = 11; –/–, n = 7. Data are presented as mean ± SEM; one-way ANOVA.

(B) Glucose tolerance test (GTT). Data are presented as mean ± SEM; two-way ANOVA; n = 8 mice per group; ****p < 0.0001.

(C) Area under the curve (AUC) analysis of the GTT shown in (B). Data are presented as mean ± SEM; one-way ANOVA.

(D) Percent change in body weight between pre-tamoxifen injection and the final day of the experiment. D707A/F, n = 5; D707A/–, n = 11; –/–, n = 8. Data are presented as mean ± SEM; one-way ANOVA.

(E) Representative H&E staining of male liver tissue.

bioRxiv preprint doi: https://doi.org/10.64898/2026.05.04.722722; this version posted May 7, 2026. The copyright holder for this preprint (which was not certified by peer review) is the author/funder, who has granted bioRxiv a license to display the preprint in perpetuity. It is made available under aCC-BY 4.0 International license.

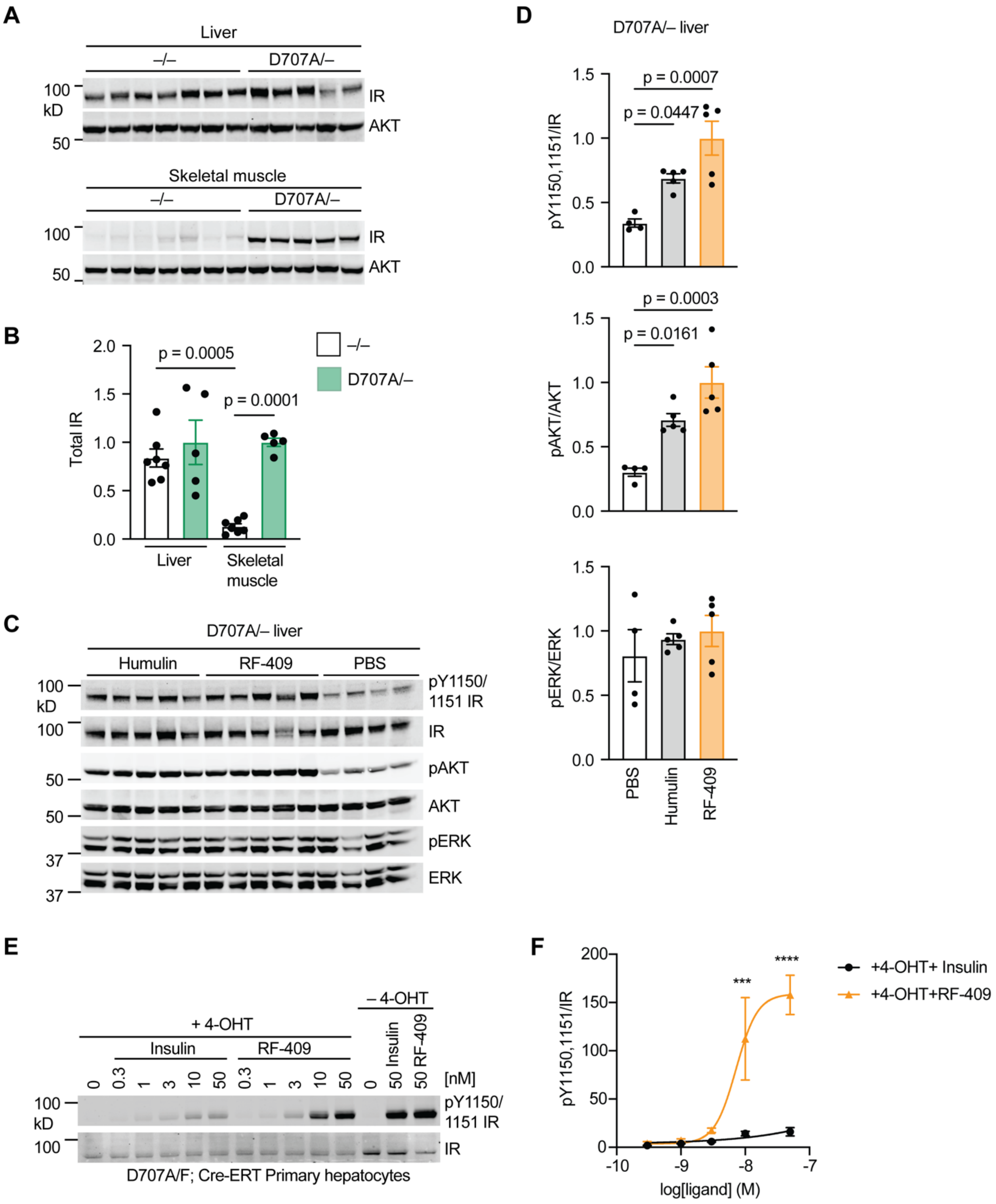

Fig. S6. RF-409 activates IR D707A in primary hepatocytes.

(A) Representative immunoblot of IR protein levels in liver and skeletal muscle from IR D707A/– and IR –/– mice following a 4 h fast.

bioRxiv preprint doi: https://doi.org/10.64898/2026.05.04.722722; this version posted May 7, 2026. The copyright holder for this preprint (which was not certified by peer review) is the author/funder, who has granted bioRxiv a license to display the preprint in perpetuity. It is made available under aCC-BY 4.0 International license.

(B) Quantification of immunoblot data shown in (A). Data are presented as mean ± SEM; IR D707A/-, *n* = 5; IR –/–, *n* = 7; one-way ANOVA.

(C) Representative immunoblot of insulin receptor (IR) autophosphorylation, AKT phosphorylation (pAKT), and ERK phosphorylation (pERK) in liver from IR D707A/– mice. Mice were fasted for 4 h and injected with PBS, Humulin (6 nmol/mouse), or RF-409 via the inferior vena cava (IVC). Liver tissue was collected 3 min after injection.

(D) Quantification of immunoblot data shown in (C). Data are presented as mean ± SEM; PBS, *n* = 4; Humulin, *n* = 5; RF-409, *n* = 5; one-way ANOVA.

(E) Representative immunoblot of IR autophosphorylation in primary hepatocytes isolated from IR D707A/F;Cre-ERT mice. Cells were treated with 4-hydroxytamoxifen (4-OHT) to delete the floxed WT IR allele and generate IR D707A/– hepatocytes, followed by stimulation with insulin or RF-409 at the indicated concentrations for 10 min.

(F) Quantification of immunoblot data shown in (E). Phosphorylation levels were normalized to total protein and expressed relative to the response to 100 nM insulin. Data are presented as mean ± SEM; *n* = 3 independent experiments; ****$p < 0.0001$.

bioRxiv preprint doi: https://doi.org/10.64898/2026.05.04.722722; this version posted May 7, 2026. The copyright holder for this preprint (which was not certified by peer review) is the author/funder, who has granted bioRxiv a license to display the preprint in perpetuity. It is made available under aCC-BY 4.0 International license.

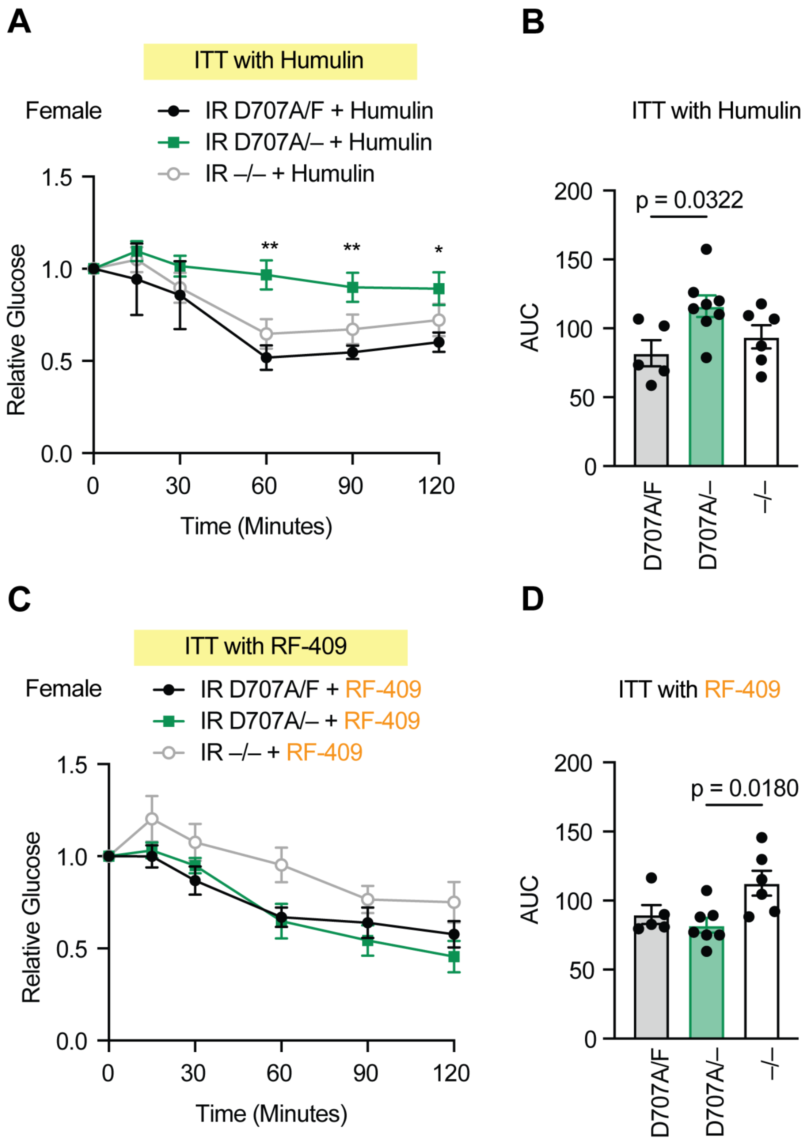

Fig. S7. RF-409, but not insulin, lowers blood glucose levels in female IR D707A mice.

(A) Insulin tolerance test (ITT) following Humulin administration in female mice. IR D707A/F, *n* = 8; IR D707A/–, *n* = 12; IR –/–, *n* = 9. Data are presented as mean ± SEM; two-way ANOVA; ***p < 0.001, ****p < 0.0001 (IR D707A/F vs. IR D707A/–).

(B) Area under the curve (AUC) analysis of the ITT shown in (A). Data are presented as mean ± SEM; one-way ANOVA.

(C) Insulin tolerance test (ITT) following RF-409 administration in female mice. IR D707A/F, *n* = 9; IR D707A/–, *n* = 11; IR –/–, *n* = 9. Data are presented as mean ± SEM; two-way ANOVA; *p < 0.05, **p < 0.01, ***p < 0.001 (IR D707A/F vs. IR D707A/–).

(D) Area under the curve (AUC) analysis of the ITT shown in (C). Data are presented as mean ± SEM; one-way ANOVA.
